# The treatment patterns, efficacy, and safety of *nab*^®^-paclitaxel for the treatment of metastatic breast cancer in the United States: results from health insurance claims analysis

**DOI:** 10.1186/s12885-015-2027-x

**Published:** 2015-12-29

**Authors:** Caihua Liang, Ling Li, Cindy Duval Fraser, Amy Ko, Deyanira Corzo, Cheryl Enger, Debra Patt

**Affiliations:** 1 Optum Epidemiology, 950 Winter Street, Suite 3800, Waltham, MA 02451 USA; 2 Celgene Corporation, 86 Morris Avenue, Summit, NJ 07901 USA; 3 Optum Epidemiology, 315 E. Eisenhower Parkway, Suite 305, Ann Arbor, MI 48108 USA; 4 McKesson Specialty Health/US Oncology, 6204 Balcones, Austin, TX 78731 USA

**Keywords:** Metastatic breast cancer, *nab*-Paclitaxel, Claims analysis

## Abstract

**Background:**

*nab*-Paclitaxel is an albumin-bound formulation of paclitaxel approved for the treatment of metastatic breast cancer (MBC). This analysis was designed to characterize the treatment patterns, efficacy, and safety of *nab*-paclitaxel for MBC treatment using health claims data from US health plans associated with Optum.

**Methods:**

Women aged ≥ 18 years who initiated *nab*-paclitaxel for MBC treatment from January 1, 2005, to September 30, 2012, and who met eligibility criteria were selected from the Optum Research Database for this analysis. Patients were required to have complete medical coverage and pharmacy benefits, ≥ 6 months of continuous enrollment, and a diagnosis of MBC prior to *nab*-paclitaxel initiation. The pattern of use for *nab*-paclitaxel (eg, regimen, schedule, duration, and administration) and claims-captured toxicities were characterized by line of therapy. Overall survival (OS) and time to next therapy or death (TNTD) were described by line of therapy, regimen, and schedule.

**Results:**

Of the 664 *nab*-paclitaxel patients, 172 (25.9 %) received it as first-line therapy, 211 (31.8 %) as second-line therapy, and 281 (42.3 %) as third-line or later therapy. Overall, the majority of patients received monotherapy (61 %) and followed a weekly (71 %) rather than an every 3 weeks treatment schedule. *nab*-Paclitaxel was often (31.7 %) combined with targeted therapy (57.5 % with bevacizumab and 23.9 % with trastuzumab or lapatinib). The median duration of therapy was 128 days (4.2 months). For the overall population, median OS was 17.4 months (22.7, 17.4, and 15.1 months in first-, second-, and third-line or later therapy, respectively). Median TNTD was 6.1 months (7.1, 6.6, and 5.3 months in first-, second-, and third-line or later therapy, respectively). For patients aged ≤ 50 years or with ≥ 3 metastatic sites, median OS was 15.6 months. No new safety signal was identified.

**Conclusions:**

In this US healthcare system, the majority of patients received *nab*-paclitaxel as second-line or later therapy, monotherapy, and weekly treatment. The efficacy and safety outcomes of *nab*-paclitaxel observed in this real-world setting appear consistent with those from clinical trial data.

## Background

Taxanes are some of the most active chemotherapeutic agents in the treatment of breast cancer [1–3]. However, sensory neuropathy, neutropenia, and significant toxicities—such as severe hypersensitivity reactions, which require substantial premedication with high doses of steroids and antihistamines—have been reported in patients treated with solvent-based (sb) taxanes (ie, paclitaxel and docetaxel) [4–6]. An albumin-bound formulation of paclitaxel (Abraxane®, *nab*-paclitaxel) was developed in an effort to overcome the toxicities associated with sb-paclitaxel and improve efficacy [7]. Preclinical studies have shown that *nab*-paclitaxel delivers a 33 % higher paclitaxel concentration to tumors and demonstrates enhanced transport across endothelial cell monolayers compared with sb-paclitaxel [7]. Recently published population pharmacokinetic data on *nab*-paclitaxel compared with sb-paclitaxel demonstrated more rapid and greater tissue penetration and slower elimination of paclitaxel [8]. *nab*-Paclitaxel was approved by the US Food and Drug Administration in January 2005 for the treatment of breast cancer after failure of combination chemotherapy, including anthracyclines, for metastatic disease or relapse within 6 months of adjuvant chemotherapy [9].

The safety and efficacy of single-agent *nab*-paclitaxel have been well established in clinical trials of patients with metastatic breast cancer (MBC) (Table 1) [10–13]. In a phase three trial [10], *nab*-paclitaxel dosed at 260 mg/m^2^ every 3 weeks (q3w) vs sb-paclitaxel dosed at 175 mg/m^2^ q3w demonstrated a significantly higher overall response rate (33 % vs 19 %; *P* = 0.001) and a significantly longer time to tumor progression (5.3 vs 3.9 months; *P* = 0.006). The incidence of grade 4 neutropenia was significantly lower with *nab*-paclitaxel compared with sb-paclitaxel. Although the incidence of grade 3 sensory neuropathy was significantly higher with *nab*-paclitaxel compared with sb-paclitaxel, it was manageable with dose modifications and treatment interruptions and improved to grade ≤ 2 in a median of 22 days. Although the 260 mg/m^2^ q3w *nab*-paclitaxel monotherapy regimen is indicated for the treatment of patients with MBC, other doses and schedules of *nab*-paclitaxel have been explored in clinical trials. In a phase two trial, three different *nab*-paclitaxel regimens (300 mg/m^2^ q3w, 100 mg/m^2^, or 150 mg/m^2^ given weekly for the first 3 of 4 weeks [qw 3/4]) were compared with docetaxel 100 mg/m^2^ q3w for the treatment of chemotherapy-naive patients with MBC [12, 13]. Results from this trial indicated that the 150 mg/m^2^ qw 3/4 dose of *nab*-paclitaxel was a significantly more effective regimen than docetaxel [13]. Median overall survival (OS) was 33.8 months compared with 22.2, 27.7, and 26.6 months for *nab*-paclitaxel 100 mg/m^2^ qw 3/4, *nab*-paclitaxel 300 mg/m^2^ q3w, and docetaxel, respectively [13]. The frequency of grade 3/4 neutropenia, febrile neutropenia, and fatigue was lower in all *nab*-paclitaxel arms compared with docetaxel. The incidence of grade 3 sensory neuropathy was higher for the 300 mg/m^2^ q3w and 150 mg/m^2^ qw 3/4 *nab*-paclitaxel regimens vs docetaxel, which may be related to the higher median dose intensities associated with these two *nab*-paclitaxel dose regimens (100 and 101 mg/m^2^/week, respectively), compared with docetaxel (33 mg/m^2^/week) [13]. The median time to improvement of sensory neuropathy to ≤ grade 2 was 20 to 22 days for *nab*-paclitaxel compared with 41 days for docetaxel [13].

**Table 1:** Select clinical trials of *nab*-P in metastatic breast cancer

Trial	Phase	Patient population	Regimen	Efficacy		Select^a^ Grade ≥ 3 AEs, %	
				PFS, mo	OS, mo	Neutropenia	Neuropathy
Monotherapy							
Ibrahim et al. 2005 [11]	2	First line (*n* = 15)	*nab*-P 300 mg/m^2^ q3w	TTP 6.1	14.6	51	11^b^
		Second line or later (*n* = 48)					
Gradishar et al. 2009 [12] & 2012 [13]	2	First line	*nab*-P 300 mg/m^2^ q3w (*n* = 76)	11.0	27.7	43	21^b^
			*nab*-P 100 mg/m^2^ qw 3/4 (*n* = 76)	12.8	22.2	25	9^b^
			*nab*-P 150 mg/m^2^ qw 3/4 (*n* = 74)	12.9	33.8	45	22^b^
Gradishar et al. 2005 [10]	3	First line(*n* = 97)	*nab*-P 260 mg/m^2^ q3w	TTP 5.3	15.0	30	10^b^
		Second line or later (*n* = 132)					
Combination therapy with cytotoxic agents							
Roy et al. 2009 [14]	2	First line (*N* = 50)	*nab*-P 125 mg/m^2^ + gemcitabine 1000 mg/m^2^ qw 2/3	7.9	Median not reached; 6-mo OS 92 %	54	8^b^
Schwartzberg et al. 2012 [15]	2	First line (*N* = 50)	*nab*-P 125 mg/m^2^ qw 2/3 + oral capecitabine 825 mg/m^2^ twice daily on days 1 and 15 of a 21-day cycle	10.6	19.9	10	2^b^
		HER2 negative					
Combination therapy with targeted agents							
Seidman et al. 2013 [16]	2	First line, HER2 negative	*nab*-P 130 mg/m^2^ qw + bev 10 mg/kg q2w (*n* = 79)	8.8	23.7	33	46
			*nab*-P 260 mg/m^2^ q2w + bev 10 mg/kg q2w (*n* = 54)	5.8	19.0	6	56
			*nab*-P 260 mg/m^2^ q3w + bev 15 mg/kg q3w (*n* = 75)	7.7	21.3	16	33
Rugo et al. 2015 [17]	3	First line, predominantly HER2 negative	*nab*-P 150 mg/m^2^ qw 3/4 + bev 10 mg/kg q2w (*n* = 271)	9.3	23.5	51	27
Mirtsching et al. 2011 [18]	2	First line (*N* = 72)	*nab*-P 125 mg/m^2^ qw 3/4 + trastuzumab 4 mg/kg bolus then 2 mg/kg qw (HER2 positive only)	14.5	29.0	11^b^	8^b^
		HER2 positive (*n* = 22)					
Yardley et al. 2013 [19]	2	First/second line (*N* = 60)	*nab*-P 125 mg/m^2^ qw 3/4 + oral lapatinib 1250 mg daily	9.1	Median not reached	22^b^	3^b^

*AE* adverse event, *bev* bevacizumab, *HER2* human epidermal growth factor receptor, *nab*-P *nab*-paclitaxel, *OS* overall survival, *PFS* progression-free survival, *TTP* time to tumor progression, *qw* every week, *q2w* every 2 weeks, *q3w* every 3 weeks, *qw 2/3* weekly for the first 2 of 3 weeks; *qw 3/4* weekly for the first 3 of 4 weeks

^a^Neutropenia and neuropathy are common grade ≥ 3 toxicities associated with *nab*-P treatment

^b^No grade 4 events

*nab*-Paclitaxel has also been studied in combination with other cytotoxic or targeted agents for the treatment of MBC (Table 1) [14–19]. Results of phase two trials of *nab-*paclitaxel in combination with gemcitabine and oral capecitabine have demonstrated efficacy and favorable tolerability. The results of other clinical trials have shown that *nab*-paclitaxel is a reasonable substitution for sb-taxanes in combination with targeted agents such as bevacizumab, trastuzumab, and lapatinib for the treatment of MBC [16–21].

Clinical trials target highly selected patients with restrictive eligibility criteria, limiting the generalizability of outcomes. Therefore, we conducted an observational study based on US health insurance claims data to characterize the therapeutic context (line of therapy, monotherapy vs combination therapy, and dosing schedule) and to estimate the OS and time to next therapy or death (TNTD) among patients who received *nab*-paclitaxel for the treatment of MBC.

## Methods

### Data source

In this retrospective cohort study, health insurance claims data were extracted from the Optum Research Database, which contains eligibility, pharmacy claims, medical claims, and other information, such as mortality data, from health plans associated with Optum. The health claims are linked to enrollment information with data covering the period from 1993 to present. The information in the claims database includes over 12 million individuals from geographically diverse locations across the United States who have both medical and pharmacy benefit coverage. Medical claims or encounter data were collected from all available healthcare sites (inpatient hospital, outpatient hospital, emergency room, physician’s office, surgery center, etc.) for virtually all types of provided services, including specialty, preventive, and office-based treatments. Diagnoses on the claims are recorded using *International Classification of Disease, Ninth Revision Clinical Modification* (ICD-9-CM) codes. Procedures map to ICD-9-CM, Current Procedural Terminology, and Healthcare Common Procedure Coding System codes. Pharmacy claims data include drug name, dosage form, drug strength, fill date, days of supply, financial information, and de-identified patient and prescriber codes, allowing for longitudinal tracking of medication refill patterns and changes in medications.

### Study population

**Fig. 1:**
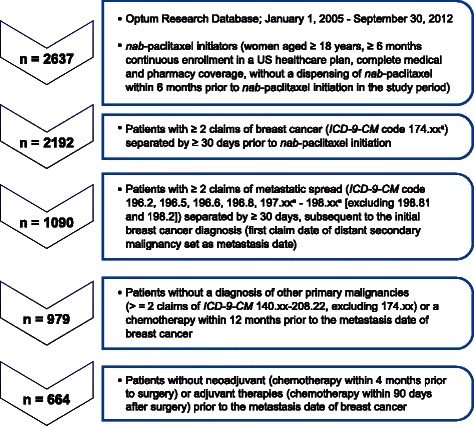
Flowchart of study patients. Patients in the Optum Research Database who met the criteria outlined in the flowchart were included in the claims analysis. ^a^xx indicates any subcode

The study population consisted of women aged ≥ 18 years with a diagnosis of MBC who received *nab*-paclitaxel treatment. Patients were eligible for the study if they had complete medical coverage and pharmacy benefits; had ≥ 6 months of continuous enrollment in a US health plan from January 1, 2005 to September 30, 2012; and had a diagnosis of MBC prior to the initiation of *nab*-paclitaxel. Patients were selected for the claims analysis based on the criteria listed in Fig. 1. Diagnosis codes appearing on claims suggesting a laboratory or diagnostic service were not considered when these criteria were applied, because these claims often reflect a “rule-out” diagnosis that has not yet been confirmed.

### Lines of therapy

New users of *nab*-paclitaxel were defined as those with a first dispensing of *nab*-paclitaxel within the study period (January 1, 2005 to September 30, 2012), with no dispensing of *nab*-paclitaxel during the 6 months prior to the first dispensing (baseline period). Patients who received neoadjuvant (≤4 months prior to surgery) or adjuvant (≤90 days after surgery) therapy with *nab*-paclitaxel were excluded. The index date was defined as the date of *nab*-paclitaxel initiation. Patients who met the cohort entry eligibility criteria were further categorized into 3 subgroups by line of therapy (first-, second-, or third-line or later) with *nab*-paclitaxel.

A patient must have received ≥ 1 cycle (defined as 30 days) of treatment prior to being defined as switching to a greater line of therapy. Any switching or addition of agents within 30 days of the start of each line of therapy was considered to be the same line of therapy.

#### First-line therapy

First-line therapy with *nab*-paclitaxel was defined as initial dispensing of *nab*-paclitaxel as the first chemotherapy received after a diagnosis of metastatic disease. All agents received within 30 days following *nab*-paclitaxel were considered part of first-line therapy.

#### Second-line therapy

Second-line therapy with *nab*-paclitaxel was defined as dispensing of *nab*-paclitaxel as part of second-line therapy, defined as additional treatment different from the first-line therapy and initiated ≥ 30 days after the first chemotherapy or after a large gap (eg, 90 days) in therapy.

#### Third-line or later therapy

Third-line or later therapy with *nab*-paclitaxel was defined as a first dispensing of *nab*-paclitaxel as part of third-line therapy, defined as any additional treatment different from any initiated first- or second-line therapy and after 60 days of the first chemotherapy or after a large gap (eg, 90 days) in therapy. Similar methods were used to identify later lines of therapy.

### Outcome identification

The study outcomes included all-cause death, TNTD, and major toxicities following *nab*-paclitaxel initiation. All-cause death was identified using Social Security Administration data linked to claims data. TNTD was used as a surrogate of progression-free survival (PFS). Major toxicities were identified following each line of therapy and determined by tabulating the 25 most frequent ICD-9-CM diagnoses codes. The toxicities of interest included select adverse events consistent with the known safety profile of *nab*-paclitaxel: neutropenia, anemia, thrombocytopenia, infections, peripheral neuropathy, asthenia, nausea, vomiting, diarrhea, fluid retention, myalgia/arthralgia, and alopecia.

### Statistical analysis

A descriptive analysis was conducted to identify the background characteristics, *nab*-paclitaxel treatment patterns, and *nab*-paclitaxel toxicities of interest in patients with MBC by line of therapy. The background characteristics, including demographics and breast cancer risk factors, were ascertained during the 6-month baseline period. Treatment patterns of *nab*-paclitaxel were described in terms of treatment regimen (monotherapy or combination therapy), treatment schedule (weekly or q3w), duration of line of therapy, number of administrations, intervals between dispensings, and dose. The occurrence of toxicity claims of interest following *nab*-paclitaxel treatment was also summarized.

A survival analysis using an intent-to-treat approach was performed to evaluate the OS and TNTD. Each patient was followed from *nab*-paclitaxel initiation in each line of therapy until the first occurrence of a study endpoint (all-cause death and TNTD, separately), disenrollment from the health plan (eg, a gap of > 32 days in membership), or the end of the study period (September 30, 2012). OS was defined as the interval between the first dispensing of *nab*-paclitaxel and death. TNTD was defined as the interval between the first dispensing of *nab*-paclitaxel and switching of line of therapy or death. Kaplan-Meier plots were used to depict the cumulative probability of OS and TNTD by line of therapy. The median OS and median TNTD as well as their 95 % CIs were also estimated. These survival analyses were conducted overall, by line of therapy, by regimen, and by schedule. A subgroup analysis was also performed among patients aged ≤ 50 years or with ≥ 3 metastatic sites.

## Results

### Patient characteristics

There were 2637 *nab*-paclitaxel initiators identified during the study period. After the eligibility criteria were applied, a total of 664 patients remained in the final analysis (Fig. 1). The 664 eligible patients were predominantly aged 50 to 69 years and were from the southern region of the United States (Table 2). There were sparse data recorded in the claims for family history of breast cancer, oral contraceptive use, hormone replacement therapy, alcohol use, obesity, and smoking. All patients had physician visits during the 6-month baseline period, 38.3 % of patients visited an emergency department, and 31.8 % of patients were admitted to a hospital. The median duration of hospitalization was 5 days (Table 2). The median length of health plan membership was 2.4 years prior to initiation of *nab*-paclitaxel.

**Table 2:** Baseline characteristics of *nab*-paclitaxel initiators by line of therapy^a^

Characteristic	First line (*n* = 172)		Second line (*n* = 211)		Third line or later (*n* = 281)		All (*N* = 664)	
	*n*	%	*n*	%	*n*	%	*n*	%
Age								
≤ 39 y	4	2.3	7	3.3	16	5.7	27	4.1
40–49 y	36	20.9	53	25.1	64	22.8	153	23.0
50–59 y	53	30.8	78	37.0	116	41.3	247	37.2
60–69 y	58	33.7	60	28.4	66	23.5	184	27.7
≥ 70 y	21	12.2	13	6.2	19	6.8	53	8.0
Geographic area								
Midwest	39	22.7	47	22.3	65	23.1	151	22.7
Northeast	12	7.0	11	5.2	25	8.9	48	7.2
South	91	52.9	123	58.3	146	52.0	360	54.2
West	30	17.4	30	14.2	45	16.0	105	15.8
Healthcare utilization								
No. of physician visits								
0	0	0	0	0	0	0	0	0
1–2	2	1.2	2	0.9	3	1.1	7	1.1
≥ 3	170	98.8	209	99.1	278	98.9	657	98.9
No. of emergency department visits								
0	103	59.9	117	55.5	190	67.6	410	61.7
1–2	51	29.7	82	38.9	72	25.6	205	30.9
≥ 3	18	10.5	12	5.7	19	6.8	49	7.4
No. of hospitalizations								
0	119	69.2	138	65.4	196	69.8	453	68.2
1–2	47	27.3	68	32.2	77	27.4	192	28.9
≥ 3	6	3.5	5	2.4	8	2.8	19	2.9
	Median	IQR	Median	IQR	Median	IQR	Median	IQR
Length of health plan membership, y	2.6	(1.4–4.2)	1.9	(1.0–3.0)	2.7	(1.7–4.0)	2.4	(1.4–3.7)
Length of inpatient stay, d^b^	5.0	(3.0–13.0)	4.0	(2.0–9.0)	5.0	(2.0–8.0)	5.0	(2.0–9.0)

*IQR* interquartile range

^a^Data are from the Optum Research Database, January 1, 2005 to September 30, 2012

^b^Among those with ≥ 1 hospital stay

### *nab*-Paclitaxel treatment patterns

Treatment patterns by line of therapy are summarized in Table 3. There were 172 (25.9 %) patients who received *nab*-paclitaxel as first-line therapy, 211 (31.8 %) as second-line therapy, and 281 (42.3 %) as third-line or later therapy. Overall, there were 405 (61.0 %) users who had *nab*-paclitaxel administered as monotherapy and 259 (39.0 %) who had *nab*-paclitaxel administered as combination therapy. When *nab*-paclitaxel was given as a combination therapy, targeted agents were often used (57.5 % bevacizumab and 23.9 % trastuzumab or lapatinib). Bevacizumab combination was more often prescribed in first-line therapy with *nab*-paclitaxel vs trastuzumab or lapatinib (81.0 vs 4.8 %). Trastuzumab combination therapy was more often given in the third line or later (39.5 %) compared with first-line (4.8 %) or second-line (17.1 %) therapy. Of the 605 users whose treatment schedules could be determined, a majority (*n* = 428 [70.7 %]) received weekly treatment and 177 (29.3 %) received q3w treatment. The median durations that patients received *nab*-paclitaxel as first-line, second-line, and third-line or later therapy were 159 days (5.2 months), 119 days (3.9 months), and 122 days (4.0 months), respectively (Table 3).

**Table 3:** Treatment patterns of *nab*-paclitaxel initiators by line of therapy^a^

Variable	First line (*n* = 172)		Second line (*n* = 211)		Third or later line (*n* = 281)		All (*N* = 664)	
	*n*	%	*n*	%	*n*	%	*n*	%
Treatment regimen								
Monotherapy	109	63.4	129	61.1	167	59.4	405	61.0
Combination chemotherapy	63	36.6	82	38.9	114	40.6	259	39.0
Bevacizumab	51	81.0	53	64.6	45	39.5	149	57.5
Trastuzumab or lapatinib	3	4.8	14	17.1	45	39.5	62	23.9
Gemcitabine, carboplatin, pegylated liposomal doxorubicin/doxorubicin, docetaxel, doxorubicin, paclitaxel, irinotecan, vinorelbine, or 5-fluorouracil	3	4.8	8	9.8	14	12.3	25	9.7
≥ 2 agents from above list	6	9.5	7	8.5	10	8.8	23	8.9
Treatment schedule^b^	152		193		260		605	
Weekly	114	75.0	134	69.4	180	69.2	428	70.7
q3w	38	25.0	59	30.6	80	30.8	177	29.3
	Median	IQR	Median	IQR	Median	IQR	Median	IQR
Duration of line treatment, d	159.0	(83.0–241.0)	119.0	(65.0–191.0)	122.0	(76.0–191.0)	128.0	(76.0–199.0)
Initial dose, unit^c^	200.0	(200.0–429.0)	200.0	(200.0–400.0)	200.0	(200.0–400.0)	200.0	(200.0–400.0)
Average dose, unit^c^	227.5	(200.0–394.4)	220.0	(200.0–400.0)	214.9	(200.0–400.0)	218.5	(200.0–400.0)

*IQR* interquartile range, *q3w* every 3 weeks

^a^Data are from the Optum Research Database, January 1, 2005 to September 30, 2012

^b^59 patients (20 in first-line, 18 in second-line, and 21 in third-line or later therapy) could not be classified into a weekly or q3w treatment schedule

^c^Each unit is equivalent to 1 mg. The dosage calculated may not reflect the exact dose dispensed or received

### *nab*-Paclitaxel safety outcomes

Table 4 shows the claims of the major toxicities of interest among patients without corresponding events during the baseline period. Anemia (26.3 %), nausea and vomiting (24.5 %), neutropenia (17.5 %), and asthenia (15.6 %) were the most common incident claims. This study also found that 14.5 % of claims were for peripheral neuropathy. These events were more frequently recorded in patients with first-line therapy compared with patients receiving *nab*-paclitaxel in later lines of therapy.

**Table 4:** Select adverse events among *nab*-paclitaxel initiators by line of therapy during the follow-up period^a,b^

Adverse event	First line (*n* = 172)			Second line (*n* = 211)			Third or later line (*n* = 281)			All (*N* = 664)		
	Total *n*^c^	*n*	%	Total *n*^c^	*n*	%	Total *n*^c^	*n*	%	Total *n*^c^	*n*	%
Neutropenia	165	33	20.0	190	26	13.7	222	42	18.9	577	101	17.5
Anemia	123	39	31.7	133	31	23.3	147	36	24.5	403	106	26.3
Thrombocytopenia	163	4	2.5	199	11	5.5	264	9	3.4	626	24	3.8
Infections	152	32	21.1	184	25	13.6	242	27	11.2	578	84	14.5
Peripheral neuropathy	129	20	15.5	155	19	12.3	200	31	15.5	484	70	14.5
Asthenia	141	28	19.9	178	25	14.0	232	33	14.2	551	86	15.6
Nausea and vomiting	143	47	32.9	159	30	18.9	179	41	22.9	481	118	24.5
Diarrhea	162	9	5.6	199	7	3.5	267	16	6.0	628	32	5.1
Fluid retention	160	9	5.6	196	12	6.1	257	16	6.2	613	37	6.0
Myalgia/arthralgia	133	14	10.5	159	20	12.6	230	17	7.4	522	51	9.8
Alopecia	171	0	0	209	3	1.4	280	5	1.8	660	8	1.2

^a^Data are from the Optum Research Database, January 1, 2005 to September 30, 2012

^b^Follow-up time was calculated from index date until disenrollment from the health plan, death (or treatment discontinuation), or the end of the study period (September 30, 2012)

^c^Total n refers to the total number of patients without baseline events for its respective subgroup

### *nab*-Paclitaxel efficacy outcomes

**Fig. 2:**
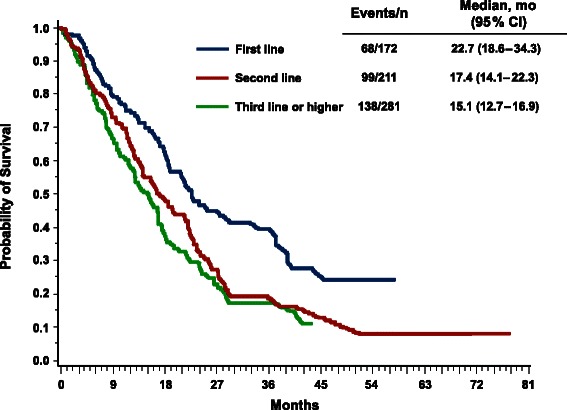
Overall survival by line of therapy. Kaplan-Meier plot depicting the cumulative probability of overall survival by line of therapy

**Fig. 3:**
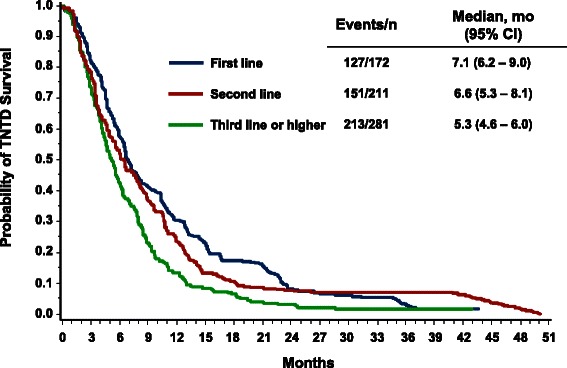
Time to next therapy or death (TNTD) by line of therapy. Kaplan-Meier plot depicting the cumulative probability of TNTD by line of therapy

Patients who received first-line *nab*-paclitaxel–based therapy appeared to have longer median survival vs second- and third-line or later therapy (Fig. 2): 22.7, 17.4, and 15.1 months, respectively (Table 5; Fig. 2). Median TNTD values were 7.1, 6.6, and 5.3 months by first-, second-, and third-line or later therapy, respectively (Table 5; Fig. 3). In the subgroup of patients aged ≤ 50 years or who had ≥ 3 metastases (*n* = 400), the median OS was 15.6 months (95 % CI, 12.9–17.4 months), and the median TNTD was 5.7 months (95 % CI, 4.9–6.4 months). Patients who received *nab*-paclitaxel combination therapy had a median survival time of 18.7 months compared with 16.8 months for those who received *nab*-paclitaxel monotherapy (Table 5); the respective values for median TNTD were 6.5 and 5.8 months.

**Table 5:** OS and TNTD among *nab*-paclitaxel initiators by line of therapy and treatment regimen^a,b^

Variable	Total no.	OS			TNTD		
		Events	Median, mo	95 % CI	Events	Median, mo	95 % CI
Overall	664	305	17.4	(16.1–19.0)	491	6.1	(5.6–6.7)
Monotherapy	405	184	16.8	(14.5–18.5)	293	5.8	(5.0–6.7)
Combination therapy	259	121	18.7	(15.9–24.0)	198	6.5	(5.8–7.8)
First line	172	68	22.7	(18.6–34.3)	127	7.1	(6.2–9.0)
Monotherapy	109	37	20.8	(16.5–39.2)	76	6.7	(5.0–7.4)
Combination therapy	63	31	23.0	(18.7–37.3)	51	8.5	(6.5–13.1)
Second line	211	99	17.4	(14.1–22.3)	151	6.6	(5.3–8.1)
Monotherapy	129	67	15.6	(12.8–21.4)	97	5.8	(4.7–7.9)
Combination therapy^c^	82	32	>18.7	–	54	7.3	(5.1–9.7)
Third or later line	281	138	15.1	(12.7–16.9)	213	5.3	(4.6–6.0)
Monotherapy	167	80	15.6	(11.6–17.8)	120	5.3	(4.2–6.1)
Combination therapy	114	58	14.3	(11.9–17.4)	93	5.5	(4.6–6.4)

*OS* overall survival, *TNTD* time to next therapy or death

^a^Data are from the Optum Research Database, January 1, 2005 to September 30, 2012

^b^Follow-up time was calculated from index date until disenrollment from the health plan, death (or treatment discontinuation), or the end of the study period (September 30, 2012)

^c^The 95 % CI of median survival time is missing because more than half of the patients survived during the study period

Median OS and TNTD values stratified by line of therapy and treatment schedule are shown in Table 6. Median OS was 18.6 months for weekly and 17.4 months for q3w *nab*-paclitaxel, and median TNTD was 6.5 months for weekly and 6.0 months for q3w *nab*-paclitaxel, respectively.

**Table 6:** OS and TNTD among *nab*-paclitaxel initiators by line of therapy and treatment schedule^a,b^

Variable	Total No.	OS			TNTD		
		Events	Median, mo	95 % CI	Events	Median, mo	95 % CI
Overall	605^c^	274	18.1	(16.7–20.8)	452	6.4	(5.8–6.8)
Weekly	428	191	18.6	(16.1–21.6)	316	6.5	(5.8–7.1)
q3w	177	83	17.4	(14.0–21.7)	136	6.0	(5.0–7.1)
First line	152	60	23.0	(19.0–37.2)	115	7.1	(6.2–9.2)
Weekly	114	49	21.6	(18.0–29.3)	85	7.4	(6.2–10.5)
q3w^d^	38	11	>22.7	–	30	6.6	(4.5–10.5)
Second line	193	86	19.4	(14.4–22.6)	138	6.9	(5.7–8.6)
Weekly	134	55	19.8	(14.4–23.8)	94	6.9	(5.7–8.8)
q3w	59	31	17.4	(12.0–26.1)	44	6.9	(5.0–9.9)
Third or later line	260	128	15.3	(12.8–17.0)	199	5.5	(4.9–6.2)
Weekly	180	87	15.9	(12.9–18.1)	137	5.8	(4.9–6.5)
q3w	80	41	13.5	(8.9–17.4)	62	5.0	(4.0–6.4)

*OS* overall survival, *q3w* every 3 weeks, *TNTD* time to next therapy or death

^a^Data are from the Optum Research Database, January 1, 2005 to September 30, 2012

^b^Follow-up time was calculated from index date until disenrollment from the health plan, death (or treatment discontinuation), or the end of the study period (September 30, 2012)

^c^59 patients (20 in first line, 18 in second line, and 21 in third or later line) could not be classified into a weekly or every 3 weeks treatment schedule

^d^The 95 % CI of median survival time is missing because more than half of the subjects survived during the study period

## Discussion

Breast cancer is a heterogeneous disease with various clinical and biological features [22]. Multiple molecular alterations and cellular pathway dysregulations may occur during disease development and progression [23]. Some types of breast cancers are more aggressive than others, and sensitivity to treatment may differ [24, 25]. To get a real-world look at *nab*-paclitaxel treatment patterns, efficacy, and safety since market approval, we carried out a claims-based retrospective analysis using a large US commercial health insurance database and selected women undergoing treatment with *nab*-paclitaxel for MBC.

Consistent with the National Comprehensive Cancer Network guidelines [1], our analysis indicated that *nab*-paclitaxel was most often prescribed as second-line or later therapy and administered as monotherapy. When *nab*-paclitaxel was used in combination, the targeted agents (e.g., bevacizumab, trastuzumab, or lapatinib) were most often prescribed. Patients treated with *nab-*paclitaxel in the first line appeared to have favorable survival relative to patients treated in later lines of therapy. However, the treatment effect of *nab*-paclitaxel as first-line therapy may have been overestimated because the criteria for first-line therapy required patients to be treated for at least 30 days. Therefore, patients who discontinued treatment early may not have been captured. Overall, the safety and efficacy profiles of *nab*-paclitaxel in this setting of US women with MBC were consistent with clinical trial experience (Table 1) [10–19].

Our analysis showed a median OS of 17.4 months for the overall population of patients with MBC who received various doses, schedules, and regimens of *nab*-paclitaxel across all lines of therapy. These results are in line with those of a phase two trial [11] and the pivotal phase three trial [10], which showed a median OS of 14.6 and 15.0 months, respectively, in patients receiving *nab*-paclitaxel monotherapy (260–300 mg/m^2^ q3w) for ≥ first-line treatment of MBC (Table 1). In a phase two trial of chemotherapy-naive patients with MBC, median OS was 22.2 to 33.8 months for weekly and 27.7 months for q3w *nab*-paclitaxel [13]. The OS claims for first-line therapy (monotherapy: median of 20.8 months; weekly median of 21.6 months) are similar to the median OS values reported in the phase two trial for the *nab*-paclitaxel 100 mg/m^2^ weekly arm (22.2 months). Notably, treatment duration with *nab*-paclitaxel was much longer in the phase two trial (6.9 months), which may account for the longer OS results vs this claims analysis.

For the patients who received *nab*-paclitaxel monotherapy, the median TNTD was 5.8 months, similar to the reported median time to disease progression of patients with MBC who received *nab*-paclitaxel monotherapy (260–300 mg/m^2^ q3w) for ≥ first-line treatment in the phase two and phase three trials: 6.1 and 5.3 months, respectively [10, 11]. However, the median TNTD in the claims analysis was shorter than the median PFS reported in the phase two trial of patients receiving first-line *nab*-paclitaxel monotherapy: median PFS of 11.1 months with 300 mg/m^2^ q3w *nab*-paclitaxel and 12.8 to 12.9 months with 100 to 150 mg/m^2^ weekly *nab*-paclitaxel [12].

This analysis also supports clinical trial data indicating that patients with poor prognostic characteristics derive a clinical benefit from *nab*-paclitaxel therapy. Patients treated with *nab*-paclitaxel who were aged ≤ 50 years or had ≥ 3 metastases had outcomes comparable with those of the overall population (median OS: 15.6 months). These results are also similar to those from a retrospective analysis of patients from the pivotal phase three trial who received therapy later than first line and had ≥ 3 metastases (median OS: 13.0 months) [26]. Furthermore, in a separate retrospective analysis of patients with poor prognosis, a favorable survival benefit was demonstrated in patients with visceral dominant metastases (OS: 15.1–32.1 months) or a short disease-free interval (OS: 14.6–19.1 months) who received *nab*-paclitaxel as first-line therapy [27]. Results from this claims analysis are similar to those for the intent-to-treat population of those trials as well (Table 1) [10, 13].

Median TNTD and OS for patients who received *nab*-paclitaxel–based combination therapy were in line with results of clinical trials of *nab*-paclitaxel–based combination therapy (Table 1) [14–19]. Our analysis showed that, when used in combination, *nab-*paclitaxel was most often given with bevacizumab (58 %), and bevacizumab combination therapy was more often initiated in the first line (81 %). It is noted that longer survival and TNTD were observed in the first line for combination therapy vs monotherapy (23.0 vs 20.8 months and 8.5 vs 6.7 months, respectively). In 2011, bevacizumab for the treatment of breast cancer was revoked by the US Food and Drug Administration [28]. The effect of this was reflected in a marked decrease (nearly 70 %) in the rate of *nab*-paclitaxel combination therapy use after 2011 and was likely due to bevacizumab being revoked (data not shown). At this time it is unclear what the optimal combination partner is for *nab*-paclitaxel in patients with human epidermal growth factor receptor 2–negative MBC. Currently a phase two/three trial is under way to determine the efficacy and safety of *nab*-paclitaxel in combination with gemcitabine or carboplatin in patients with triple-negative MBC [29].

The common adverse events identified in the clinical trials were also explored in this claims-based study. The occurrence of select known *nab*-paclitaxel toxicities (eg, neutropenia, peripheral neuropathy, anemia, infections, and nausea and vomiting) ranged from 15 % to 26 % and was lower than that noted in clinical trials (Table 1) [10–19]. In particular, the frequency of reported neuropathy was relatively low (<15 %) compared with that reported in clinical trials for *nab*-paclitaxel [10–12], indicating that this adverse event may have been underrepresented in the claims database. This is likely explained in part by the more robust patient monitoring and collection of safety data in the clinical trial setting. In addition, claims data for analysis tend to bias toward underreporting in comparison with prospective National Cancer Institute Common Terminology Criteria for Adverse Events documentation.

Although claims analyses are extremely valuable for the efficient and effective examination of healthcare outcomes, treatment patterns, and healthcare resource utilization, it is challenging to compare our study findings with those from clinical trials. Historical trials of *nab*-paclitaxel recruited patients according to highly restrictive criteria, and the patients received a specific line of *nab*-paclitaxel therapy, treatment regimen, or treatment schedule during the study period. For example, the phase two trials often targeted first-line therapy with various doses and schedules, whereas the pivotal phase three trial mixed lines of therapy at a q3w dose/schedule (Table 1). In addition, claims analyses are unable to estimate disease progression. TNTD may be perceived as a weak surrogate for PFS because a potential time lapse between disease progression and initiation of a new line of therapy is not captured. This in effect could overestimate a benefit of treatment. Estimating an overall response rate and determining the grade of toxicities are also not feasible using claims data.

Furthermore, because claims are collected for the purpose of payment and not research, inherent limitations in our claims analysis included the potential for incorrect reporting of diagnosis codes, mixing patients with early-stage breast cancer with patients with MBC, missing information on hormone receptor status, misinterpreting disease-onset dates, misclassifying the line of therapy, and inaccurately estimating actual drug dosages and schedules. However, the application of a well-defined algorithm, including the combination of diagnoses, procedures, and medications, reduced the potential for false-positive cases and the misclassification of line of therapy.

## Conclusions

*nab*-Paclitaxel is administered more frequently as a single agent on a weekly schedule and as second-line or later therapy to patients with MBC in a US healthcare system. This analysis demonstrates the use of *nab*-paclitaxel weekly or q3w and its use for the treatment of patients aged ≤ 50 years or with ≥ 3 metastatic sites. The benefit observed in this US healthcare system is consistent with that from previously reported clinical trials. No new safety signals were identified. Furthermore, our analysis showed that, when used in combination, *nab*-paclitaxel was most often combined with bevacizumab in first-line therapy. However, because the accelerated approval of bevacizumab for MBC was withdrawn due to the lack of an OS advantage in the RIBBON-1 and AVADO trials [28], bevacizumab is no longer used as standard therapy in MBC. Additional *nab*-paclitaxel combination partners are being evaluated in patients with MBC, including gemcitabine or carboplatin in patients with triple-negative MBC [29]. Identification of an optimal *nab*-paclitaxel combination regimen may provide additional options for patients with MBC. Finally, outcomes of this real-world claims analysis are consistent with the data demonstrated in key clinical trials, affirming the effectiveness and manageable safety profile of *nab*-paclitaxel across all lines of therapy in patients with MBC.

## Abbreviations

ICD-9-CM: International Classification of Diseases, Ninth Revision, Clinical Modification
MBC: Metastatic breast cancer
OS: Overall survival
PFS: Progression-free survival
q2w: Every 2 weeks
q3w: Every 3 weeks
qw 2/3: Weekly for the first 2 of 3 weeks
qw 3/4: Weekly for the first 3 of 4 weeks
qw: Weekly
sb: Solvent based
TNTD: Time to next therapy or death
